# Applicability of 14 Formulas for Estimating Glomerular Filtration Rate in the Evaluation of Renal Function before and after Nephron-Sparing Surgery in Patients with Renal Tumors

**DOI:** 10.1155/2022/3330442

**Published:** 2022-05-09

**Authors:** Qiuyan Li, ZeSong Yang, Shiwen Zheng, Yangbiao Wu, Wanghai Cai, Minxiong Hu, Qingguo Zhu, Liefu Ye

**Affiliations:** ^1^Shengli Clinical Medical College of Fujian Medical University and Department of Urology, Fujian Provincial Hospital, Fuzhou 350001, China; ^2^Medical College, Anhui University of Science and Technology, Huainan 232000, China

## Abstract

To compare the applicability of 14 equations of estimating glomerular filtration rate (eGFR) before and after nephron-sparing surgery (NSS) for renal function assessment of patients with renal tumors. Preoperative and postoperative GFR is measured by emission computed tomography (ECT) with 99mTc-DTPA as an imaging agent as reference GFR (rGFR) to compare with all formulas. Spearman correlation analysis and Bland–Altman agreement analysis were used to evaluate the correlation between rGFR and eGFR1 to 14 before and after surgery. A total of 50 cases including 22 males and 28 females were included. The results of preoperative eGFR1–14 correlated with rGFR (*P* < 0.05). The calculation results of all estimation formulas have a significant correlation with preoperative GFR. Preoperative MDRD-I, CKD-EPI _SCysC_, and FAS _Scr-SCysC_ have good consistency. The CG formula has the highest precision and FAS _Scr-SCysC_ has the highest accuracy. A total of 30 patients followed up after surgery, and postoperative rGFR correlated with CG, CKD-EPI, FAS, and BIS formulas (*P* < 0.05). But postoperative rGFR has no significant correlation with MDRD and Schwartz (*P* > 0.05). Postoperative CKD-EPI _Scr-SCysC_ has best consistency, and FAS _Scr-SCysC_ has the highest accuracy and precision. Our data suggest that eGFR equations evaluated by both serum creatinine (Scr) and cystatin C (SCysC) is not necessarily better than those evaluated by one of them alone. Among all enrolled equations, FAS _Scr-SCysC_ is the best one to evaluate postoperative GFR in patients with renal tumors.

## 1. Introduction

GFR refers to the number of milliliters of plasma that the kidneys completely filter and clear a certain metabolite in plasma within a unit time [1]. It is an important indicator for effectively evaluating the renal function of patients [2]. Methods based on the clearance of exogenous markers, like inulin, 51Cr-EDTA, and iohexol, are the gold standard for glomerular filtration rate (GFR) measurement, but are cumbersome and infrequently used in clinical settings [3]. At present, the clinically accurate determination of GFR mainly relies on ECT, with proven ease of operation and good repeatability [4]. In clinical work, relying on the biochemical results of renal function in the laboratory, such as Scr, blood urea nitrogen (BUN), and SCysC, calculating GFR through formulas is more convenient, easy, economical, and less harmful to patients [5].

Since the Cockcroft-Gault (CG) formula was widely used in clinical practice in 1976 [6], eGFR formulas based on Scr and SCysC have been developed endlessly. The National Kidney Foundation (NKF) guidelines recommend the use of the CG formula, MDRD (modification of diet in renal disease) formula, and CKD-EPI (chronic kidney disease epidemiology collaboration) formula [7], but the results of the current studies have not confirmed that the above recommended formula is the optimal one. Studies have shown that Scr-based CKD-EPI has better applicability than MDRD [8, 9]. The research results of Liu et al. on adult CKD patients in China show that the CG formula has better applicability [10]. At present, the commonly used formulas in clinical practice are FAS (full age spectrum), MDRD-I, MDRD -II, MDRD-China (MDRD-C), CKD-EPI (Scr), BIS-1 formula, Schwartz's formula, and so on. Estimated glomerular filtration rate (eGFR) is prone to bias in specific situations or special populations [11]. So far, there are few studies in China that systematically evaluate the difference in eGFR calculation results of patients with kidney tumors before and after nephron-sparing surgery. We used all 14 estimating formulas to calculate the GFR of renal tumor patients undergoing nephron-sparing surgery to verify the applicability of various formulas to renal tumor patients in my country.

## 2. Materials and Methods

### 2.1. Sources of Data and Measurements

A total of 50 patients with renal tumor who underwent NSS in Fujian Provincial Hospital from July 2020 to July 2021 were enrolled. All patients had no renal puncture, interventional surgery, radiotherapy, or chemotherapy, or other antitumor-related treatments before surgery. No history of CKD disease, renal trauma, and surgery. Clinical data include gender, age, height, weight, preoperative creatinine, urea, nitrogen, albumin (Alb), cystatin C, rGFR measured by ECT, and pathological diagnosis, as given in Table 1.

The patient's serum creatinine concentration was measured with the alkaline picric acid method using the Beckman CX3 analyzer. Serum cystatin C was measured on the Beckman UniCel DxC800 biochemical analyzer with the latex enhanced immunoturbidimetric method.

99mTc-DTPA imaging determination rGFR: before the examination, the patient drinks 300–500 ml of water, empties the bladder, and takes the supine position. The probe is placed on the back waist, and the field of view includes both the kidneys and bladder. The imaging agent is injected into the vein of one side of the elbow in the form of a “bullet,” and two sets of images of blood perfusion and renal function are dynamically collected in two phases. The acquisition field includes the kidneys, ureters, and bladder. The first phase is 1 s/frame, and 60 frames are collected. The second phase is 20 s/frame, and the collection is 29 minutes. After the collection is completed, the empty syringe is collected again and counted for 6 s. Calculate the dose of drug injected into the body according to the count value of the syringe before and after. Use the ROI technology to outline the outline and background area of the kidneys. Then, use the supporting software to process the image and generate the kidney map to obtain the GFR of the kidneys.

### 2.2. Statistical Analysis

The measured height, weight, Scr, BUN, Alb, and SCysC values were, respectively, substituted into the 14 estimation equations included in this study, and eGFR1–14 were calculated, as given in Table 2. Using SPSS 16.0 and MedCalc 15.2.2 statistical software, perform analysis. Spearman correlation analysis and Bland–Altman agreement analysis were used to evaluate the correlation between rGFR and eGFR1–14 before and after surgery.

Accuracy means the degree to which the measured value of the formula is consistent with rGFR, indicating the correctness of the calculation result. The greater the number of close agreement between the eGFR results and rGFR values of multiple patients calculated by each formula, the higher the precision of the results, showing the repeatability and stability of the formula.

## 3. Results

A total of 50 cases including 22 males and 28 females, aged 17−80 years old, with an average of 53.8 ± 10.2 years old (male), 50 ± 10.5 years old (female). The average preoperative rGFR of 50 patients was 90.04 ± 15.42 (male) and 91.71 ± 19.67 (female). Only 3 patients had rGFR less than 60 ml/min/1.73 m^−2^ and no patients had rGFR less than 30 ml/min/1.73 m^−2^. Of the 30 patients followed up after surgery, there were 12 males with an average of 56.1 ± 13.1 years old and 18 females with 48.8 ± 11.2 years old. The average time from surgery to reexamination of rGFR for 30 patients was 262 days. The average postoperative rGFR of 30 patients was 93.94 ± 20.26 (male) and 93.49 ± 22.62 (female).

### 3.1. Correlation Analysis of Preoperative eGFR and rGFR

RGFR is correlated with all preoperative GFR calculated by each formula (*P* < 0.05), among which eGFR1 (CG formula), eGFR6 (CKD-EPI _Scr_), eGFR7 (CKD-EPI _SCysC_), eGFR8 (CKD-EPI _Scr-SCysC_), eGFR9 (Schwartz _Scr-SCysC_), eGFR10 (FAS_Scr_), eGFR11 (2017 FAS _SCysC_), eGFR12 (FAS _Scr-SCysC_), eGFR13 (BIS-1_Scr_), and eGFR14 (BIS-2 _Scr-SCysC_) have a significant correlation(*P* ≤ 0.001), as given in Table 3.

### 3.2. Consistency Analysis of Preoperative eGFR and rGFR

**Figure 1:**
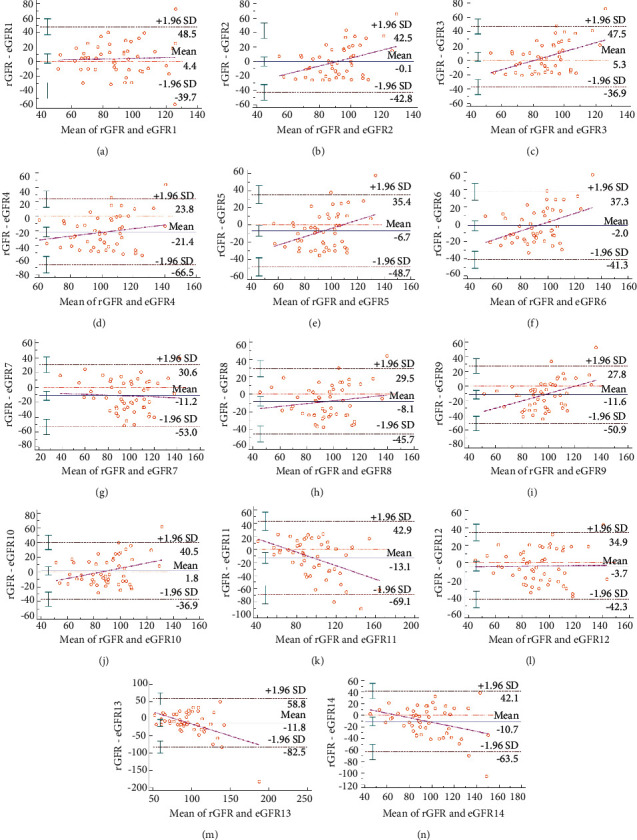
Bland–Altman plot of preoperative eGFR and rGFR in 50 patients.

The mean line of eGFR2 (MDRD-I) is closest to 0, indicating that its calculation result is closest to rGFR, as shown in Figure 1.

The closest moving average value to 0 is −0.13 (B). Only one calculated eGFR value was outside the 95% CI (B, G, L). The absolute minimum value of the intercept width is −0.2096 (A). The absolute value of the slope is the smallest, which is −0.0144 (L). The absolute value of the slope is the largest at −0.7026 (M).

Followed by eGFR10 (FAS _Scr_), the average line is only 1.8. The eGFR2 (MDRD-I), eGFR7 (CKD-EPI _SCysC_), and eGFR12 (FAS _Scr-SCysC_) have good consistency, and only one patient's GFR is outside the 95% confidence interval (95% CI).

Among the 14 formulas, the absolute value of the intercept width of eGFR1 (CG formula) is the smallest, which is −0.2096, indicating that eGFR1 (CG formula) has highest accuracy than other formulas. Followed by eGFR12 (FAS _Scr-SCysC_) and eGFR7 (CKD-EPI _SCysC_) are −5.0456 and −5.4934 respectively. The minimum absolute value of the slope of eGFR12 (FAS _Scr-SCysC_) is −0.0144, indicating the highest accuracy. Followed by eGFR1 (CG formula) and eGFR7 (CKD-EPI _SCysC_), they are −0.0516 and −0.0592, respectively. Among the CKD-EPI related formulas (eGFR6, eGFR7, and eGFR8), the cystatin C-based formula has the highest consistency than the equation based on creatinine and the combination formula. The FAS _Scr-SCysC_ formula has the highest consistency than those based on creatinine or cystatin C alone.

### 3.3. Correlation Analysis of Postoperative eGFR and rGFR

The postoperative GFR of 30 patients measured in 14 formulas was analyzed by Spearman correlation with rGFR. The results are given in Table 4. The rGFR is correlated with eGFR1 (CG formula), eGFR6 (CKD-EPI _Scr_), eGFR7 (CKD-EPI _SCysC_), eGFR8 (CKD-EPI _Scr-SCysC_), eGFR10 (FAS _Scr_), eGFR11 (2017 FAS _SCysC_), eGFR12 (FAS _Scr-SCysC_), eGFR13 (BIS-1 _Scr_), and eGFR14 (BIS-2 _Scr-SCysC_) (*P* < 0.05). But rGFR has no significant correlation with eGFR2 (MDRD-I), eGFR3 (MDRD-II), eGFR4 (MDRD-C), eGFR5 (MDRD), and eGFR9 (Schwartz _Scr-SCysC_) (*P* > 0.05).

### 3.4. Consistency Analysis of Postoperative eGFR and rGFR

Among eGFR1–14, the absolute value of the arithmetic mean of eGFR8 (CKD-EPI _Scr-SCysC_) is closest to 0. Then, there are eGFR14 (BIS-2 _Scr-SCysC_), eGFR5 (MDRD), and eGFR11 (2017 FAS _SCysC_), which have good consistency for arithmetic mean less than 2. The arithmetic mean of eGFR4 (MDRD-C) is 17.1, which is the largest value among the 14 formulas.

All calculated GFR values of eGFR8 (CKD-EPI _Scr-SCysC_) are within 95% CI means it has better consistency. Except for eGFR4 (MDRD-C) and eGFR10 (FAS _Scr_), which have two GFR values outside the 95% CI, there is only one other formula. Among the 14 formulas, the absolute value of the slope and intercept of eGFR12 (FAS _Scr-SCysC_) is the smallest, indicating that its accuracy and precision are the highest. The accuracy of eGFR4 (MDRD-C), eGFR7 (CKD-EPI _SCysC_), eGFR10 (FAS _Scr_), and eGFR1 (CG formula) is higher, for the absolute value of their slopes are all less than 0.1. The absolute values of the intercepts of eGFR10 (FAS _Scr_) and eGFR7 (CKD-EPI _SCysC_) are smaller than those of other formulas, indicating that the accuracy of these two formulas is higher than others, as given in Table 4.

## 4. Discussion

In recent years, with the development of early screening for malignant tumors and the improvement of people's health awareness, the early diagnosis rate of renal tumors has increased significantly. The correct assessment of renal function is of great importance to decide surgical strategy. GFR is a direct indicator for evaluating renal function. GFR measurement of the patients with renal tumor before and after nephron-sparing surgery may be important in treatment strategy decision and prognosis evaluation. The removal rate of inulin is an ideal method for measuring GFR, but procurement of inulin and the cumbersome procedure pose a challenge for its routine clinical use. Among many GFR estimation formulas, how to choose an appropriate formula is a puzzled problem faced by clinicians. In recent years, most of the eGFR evaluation equations have been verified by patients with chronic kidney disease, and the eGFR formula is rarely used in the evaluation of renal function in patients with renal tumors. We use the 99mTc-DTPA renal function imaging method to measure GFR as the reference standard (rGFR) [22] to assess the applicability of 14 commonly used formulas in patients with renal tumors.

The results of the study showed that there was a significant correlation between the GFR calculated by the 14 estimation formulas and rGFR before the surgery. Studies have shown that the combined formula of serum creatinine and cystatin C is better than using one of them alone [23, 24]. Among the 14 formulas included in this study, the calculation results of the three formulas including MDRD-I, CG formula, CKD-EPI _SCysC_, and FAS _Scr-SCysC_ are in good agreement with rGFR. Among them, the CG formula has the highest precision, and FAS _Scr-SCysC_ has the highest accuracy. Renal tumors can occur in almost all age groups [25]. The FAS formula is suitable for patients of all ages and has good applicability for preoperative renal function assessment [19].

In the results calculated after the operation, 5 formulas had no significant correlation (*P* > 0.05), including MDRD-I, MDRD-II, MDRD-C, MDRD, and Schwartz _Scr-SCysC_. These formulas have limitations for the evaluation of renal function in patients with renal tumors after surgery. The Schwartz formula is mainly used to evaluate the renal function of children with CKD [18]. The youngest age of the patients in this study is 17 years old. Therefore, it is verified that the Schwartz formula is not highly applicable to patients over 14 years of age. In the evaluation of renal function in adult patients with renal tumors after surgery, this method is not recommended.

In 1999, the US MDRD research team found that age, gender, and race were independent variables of GFR and derived the original MDRD formula [15]. In 2000 and 2007, MDRD-I and MDRD-II were obtained by improving the formula [12, 13]. In 2006, the Chinese eGFR project coworker group researched and imitated the development process of the MDRD formula to arrive at the improved c-MDRD formula [14]. In our study, the calculation results of the four MDRD correlation formulas have no significant correlation with rGFR, indicating that they are not suitable for eGFR calculations for postoperative patients. The National Kidney Foundation (NKF) and the American Society of Nephrology (ASN) established a joint working group in 2020 to evaluate the eGFR estimation formula [26]. On November 4, 2021, NEJM officially published the results of two research teams on eGFR estimation of race coefficient. The results of these two studies show that the eGFR estimation formula based on serum cystatin C is more accurate than the estimation formula based on serum creatinine [27]. So, they recommended the following: the race coefficient has limited influence on the eGFR calculation, and the race coefficient can be removed; each clinical laboratory should try its best to detect serum cystatin C; and further research to find better markers for eGFR estimation [26, 27]. The population included in the MDRD study is mainly concentrated in patients aged 18–70 [15]. Past studies have shown that when patients have low GFR, the MDRD simplified formula is less accurate and tends to underestimate renal function [28]. The renal function of patients after renal surgery has declined to a certain extent. Therefore, the MDRD formula is not suitable for postoperative renal function assessment. But, we should not ignore the possibility of errors caused by the reduction in sample size.

In 2016, Pottel et al. proposed an eGFR equation based on Scr/Q_Scr_ that is applicable to all-year-olds (full age spectrum, FAS), where Q_Scr_ is the median number of Scr for healthy people of a specific age and gender [19]. In 2017, the research group used cystatin C instead of Scr and proposed the FAS _SCysC_ formula and the FAS _Scr-SCysC_ formula [20]. Studies on the applicability of the FAS formula in the Chinese population have confirmed that FAS _Scr-SCysC_ has higher applicability in China [29]. In this study, GFR calculated by FAS _Scr-SCysC_ formula after surgery has the highest accuracy and precision. Patients after surgery are recommended to use this equation for renal function assessment.

In 2009, the United States Chronic Kidney Disease Epidemiology Cooperative Group proposed the CKD-EPI _Scr_ formula using Scr, age, gender, and race as calculation variables [16]. In 2012, the CKD-EPI research group proposed the CKD-EPI _SCysC_ formula and the CKD-EPI _Scr-SCysC_ formula based on standardized Scr and SCysC [17]. In the comparison and verification of the three CKD-EPI formulas, it is found that the combined formula has no significant difference in calculation deviation compared with the two separate formulas, but the precision and diagnostic accuracy are higher [30]. Our results show that the calculation results of the CKD-EPI _Scr-SCysC_ formula are highly consistent, and the postoperative GFR calculated by CKD-EPI _SCysC_ is more accurate than the other two formulas. These reflect the advantage of cystatin C in early recognition of renal function damage. CKD-EPI formula is mainly derived and established based on CKD patients as the baseline data. The applicability of the formula is different because of the significant differences in renal pathology between patients with renal cancer and CKD.

The BIS-1-2 formula is based on a study of elderly CKD white patients over 70 years of age [21]. In this study, neither preoperative nor postoperative renal function assessment has good applicability.

## 5. Conclusion

For renal function assessment in patients with renal tumors before surgery, eGFR1–14 are all significantly correlated. Among them, the MDRD-I formula can more accurately estimate the GFR of patients. In similar formulas, there is no accurate conclusion about which is better for creatinine or cystatin. In the CKD-EPI formula, the formula of using cystatin C alone is more consistent. While in the FAS formula, the combination of the two is required to calculate GFR more accurately. After surgery, the MDRD-related formula and Schwartz _Scr-SCysC_ are not applicable in the calculation of glomerular filtration rate. Among them, CKD-EPI _Scr-SCysC_ has good consistency in the assessment of postoperative GFR in patients with renal cancer. The FAS _Scr-SCysC_ formula has the highest accuracy and precision. Both of these are recommended for clinical use. However, in this study, due to the small number of postoperative patients, large-scale and sample size clinical studies can still be carried out in the future to confirm it.

## Figures and Tables

**Table 1:** Basic characteristics of the 50 patients.

Characteristic (*n* = 50)	Male	Female
*N*	22	28
Age (years)	17–80	29–73
Average age (years)	53.8 ± 10.2	50.0 ± 10.5
Height (cm)	171.6 ± 6.1	159.0 ± 4.1
Weight (kg)	70.4 ± 9.2	57.4 ± 6.6
Plasma creatinine (mg/dl)	0.94 ± 0.086	0.75 ± 0.089
Plasma urea nitrogen (mg/dl)	98.9 ± 21.5	91.1 ± 18.5
Plasma albumin (g/dl)	440.0 ± 20.0	433.2 ± 26.3
rGFR (ml/min/1.73 m^−2^)	90.04 ± 15.42	91.72 ± 19.67
Tumor size (cm)	3.3 (1.3–10.5)	4.1 (0.8–8.0)

rGFR category		
rGFR ≥ 90	10	14
60 ≤ rGFR < 90	12	11
rGFR < 60	—	3

Pathologic type		
Angiomyolipoma (AML)	3	11
chRCC	2	—
ccRCC	16	13
Mucinous tubular and spindle cell carcinoma	—	1
ESC RCC	1	—
RNET	—	1
Others	—	2

RGFR, reference glomerular filtration rate; tumor size, the maximum diameter of the tumor; AML, angiomyolipoma; chRCC, chromophobe renal cell carcinoma; ccRCC, clear cell renal cell carcinoma; ESC RCC, eosinophilic solid and cystic renal cell carcinoma; RNET, renal neuroendocrine tumor.

**Table 2:** 14 formulas for calculating the glomerular filtration rate.

eGFR1	CG formula [6]: (0.85 if female) × (140−age) × bodyweight/(72 × Scr)

eGFR2	MDRD (simplified) I [12]: 186 × Scr^−1.154^ × age^−0.203^ × (0.742 if female)
eGFR3	MDRD (simplified) II [13]: 175 × Scr^−1.154^ × age^−0.203^ × (0.742 if female)
eGFR4	MDRD-C (for Chinese) [14]: 186 × Scr^−1.154^ × age^−0.203^ × (0.742 if female) × (1.233 if Chinese)
eGFR5	MDRD formula [15]: 170 × Scr^−0.199^ × age^−0.176^ × BUN^−0.17^ × Alb^0.138^ × (0.762 if female)
eGFR6	CKD-EPI _Scr_ [16]: *a* × (Scr/b)^c^ × 0.399^age^
	*a* = 144 (if female), *a* = 141 (if male)
	*b* = 0.7 (if female), *b* = 0.9 (if male)
	*c* = −0.329 when Scr ≤ 0.7 mg/dL (if female); *c* = −1.209 when Scr ＞ 0.7 mg/dL (if female); *c* = −0.441 when Scr ≤ 0.9 mg/dL (if male); *c* = −1.209 when Scr ＞ 0.9 mg/dL (if male)
eGFR7	CKD-EPI_SCysC_ [17]: 133 × (SCysC/0.8)^a^ × (0.996)^age^ × (0.932 if female); *a* = −0.499 when SCysC ≤ 0.8 mg/l; *a* = −1.328 when SCysC ＞ 0.8 mg/L
eGFR8	CKD-EPI_Scr-SCysC_ [17]: *a* × (Scr/b)^c^ × (SCysC/0.8)^d^ × (0.995)^age^
	*a* = 130 (if female), *a* = 135 (if male)
	*b* = 0.7 (if female), *b* = 0.9 (if male)
	*c* = −0.248 when Scr ≤ 0.7 mg/dL (if female); *c* = −0.601 when Scr ＞ 0.7 mg/dL (if female); *c* = −0.207 when Scr ≤ 0.9 mg/dL (if male); *c* = −0.601 when Scr ＞ 0.9 mg/dL (if male)
	*d* = −0.375 when SCysC ≤ 0.8 mg/L; *d* = −0.711 when SCysC ＞ 0.8 mg/L
eGFR9	Schwartz_Scr-SCysC_ [18]: 39.8 × (height/Scr)^0.456^ × (1.8/SCysC)^0.418^ × (30/BUN)^0.079^ × (1.076 if male) × (height/1.4)^0.179^
eGFR10	FAS_Scr_ [19]: 107.3/(Scr/QScr) when 2 ≤ age ≤ 40
	0.988 (age^−40^) × 107.3/(Scr/QScr) when age ＞ 40
	QScr = 0.7 mg/dL (if female); QScr = 0.9 mg/dL (if male)
eGFR11	FAS_SCysC_ [20]: 107.3/(SCysC/Q_SCysC_) when 2 ≤ age ≤ 40
	0.988 (age^−40^) × 107.3/(SCysC/Q_SCysC_) when age＞40
	*Q*_SCysC_ = 0.82 when age ＜ 70; *Q*_SCysC_ = 0.95 when age ≥ 70
eGFR12	FAS_Scr-SCysC_ [20]: 0.988 (age^−40^) × 107.3/[*α* × Scr/Q_Scr_ ＋ (1 − *α*) × SCysC/Q_SCysC_] when age＞40
	*Q*_SCysC_ = 0.82 when age＜70; *Q*_SCysC_ = 0.95 when age ≥ 70 *α* = 0.5
eGFR13	BIS − 1 _Scr_ [21]: 3736 × Scr^−0.87^ × age^−0.95^ × (0.82 if female)
eGFR14	BIS − 2 _Scr-SCysC_ [21]:767 × SCysC^−0.61^ × Scr^−0.40^ × age^−0.57^ × (0.87 if female)

**Table 3:** Spearman correlation analysis of 14 evaluation formulas for preoperative GFR.

Formulas	eGFR (ml/min/1.73 m^−2^)	Spearman's correlation	
		*r*	*P*
rGFR	90.98 ± 22.90	—	—
eGFR1	86.61 ± 22.03	0.564	0.000
eGFR2	91.11 ± 15.42	0.413	0.003
eGFR3	85.72 ± 14.51	0.413	0.003
eGFR4	112.34 ± 19.01	0.413	0.003
eGFR5	97.64 ± 15.97	0.421	0.002
eGFR6	92.96 ± 15.49	0.524	0.000
eGFR7	102.19 ± 24.00	0.541	0.000
eGFR8	99.05 ± 10.05	0.561	0.000
eGFR9	102.54 ± 15.60	0.455	0.001
eGFR10	89.18 ± 17.41	0.547	0.000
eGFR11	104.05 ± 34.59	0.555	0.000
eGFR12	94.69 ± 22.64	0.590	0.000
eGFR13	102.83 ± 38.33	0.548	0.000
eGFR14	101.68 ± 31.10	0.586	0.000

Comparison of the correlation between eGFR and rGFR of each formula, *P* < 0.05.

**Table 4:** Spearman correlation analysis and Bland–Altman agreement analysis of 14 eGFR and rGFR after surgery.

Formula	eGFR	Spearman's correlation		Arithmetic mean	Slope	Intercept	95% CI (*n* (%))
		R	*P*				
rGFR	93.67 ± 22.08	—	—	—	—	—	—
eGFR1	87.92 ± 23.51	0.499	0.005	−5.7262	0.08306	−13.2684	29/30
eGFR2	89.97 ± 18.30	0.305	^*∗*^0.101	−3.7002	−0.2787	21.8890	29/30
eGFR3	84.65 ± 17.22	0.305	^*∗*^0.101	−9.0210	−0.3672	23.7211	29/30
eGFR4	110.93 ± 22.57	0.305	^*∗*^0.101	17.2630	0.03263	13.9253	28/30
eGFR5	94.77 ± 19.47	0.324	^*∗*^0.081	1.0985	−0.1824	18.2889	29/30
eGFR6	91.49 ± 17.38	0.421	0.021	−2.1787	−0.3217	27.6003	29/30
eGFR7	95.27 ± 23.23	0.444	0.014	1.6039	0.06294	−4.3426	29/30
eGFR8	94.02 ± 19.33	0.471	0.009	0.3488	−0.1644	15.7741	30/30
eGFR9	97.34 ± 14.49	0.284	^*∗*^0.128	3.6656	−0.5510	56.2854	29/30
eGFR10	88.86 ± 21.03	0.480	0.007	−4.8075	−0.06502	1.1269	28/30
eGFR11	95.11 ± 27.50	0.511	0.004	1.4373	0.2681	−23.8695	29/30
eGFR12	90.69 ± 21.65	0.537	0.002	−2.9858	−0.02436	−0.7408	29/30
eGFR13	100.77 ± 34.90	0.581	0.001	7.0974	0.5963	−50.8760	29/30
eGFR14	94.74 ± 24.52	0.582	0.001	1.0664	0.1288	−11.0627	29/30

^*∗*^ *P* > 0.05, no significant correlation；95% CI, numbers within 95% CI.

## Data Availability

The data used to support the findings of this study are available from the corresponding author upon request.
